# A Method for Cheating Indication in Unproctored On-Line Exams

**DOI:** 10.3390/s22020654

**Published:** 2022-01-15

**Authors:** Dan Komosny, Saeed Ur Rehman

**Affiliations:** 1 Department of Telecommunications, Brno University of Technology, 616 00 Brno, Czech Republic; 2 College of Science and Engineering, Flinders University, Adelaide 5042, Australia; saeed.rehman@flinders.edu.au

**Keywords:** network, end device, location, IP address, cheating, e-learning, exam, Moodle, COVID-19, lockdown

## Abstract

COVID-19 has disrupted every field of life and education is not immune to it. Student learning and examinations moved on-line on a few weeks notice, which has created a large workload for academics to grade the assessments and manually detect students’ dishonesty. In this paper, we propose a method to automatically indicate cheating in unproctored on-line exams, when somebody else other than the legitimate student takes the exam. The method is based on the analysis of the student’s on-line traces, which are logged by distance education systems. We work with customized IP geolocation and other data to derive the student’s cheating risk score. We apply the method to approx. 3600 students in 22 courses, where the partial or final on-line exams were unproctored. The found cheating risk scores are presented along with examples of indicated cheatings. The method can be used to select students for knowledge re-validation, or to compare student cheating across courses, age groups, countries, and universities. We compared student cheating risk scores between four academic terms, including two terms of university closure due to COVID-19.

## 1. Introduction

Educational institutions have broadly implemented distance learning, especially during the COVID-19 pandemic. Despite its advantages, a common problem is the unproctored on-line examination where cheating is easier than in proctored written or oral exams. Currently, there are no definite solutions available to combat the problem of cheating. In this work, we elaborate a method to indicate that a student cheated in the form that she/he shared their login credentials with somebody else, who actually took the exam (a better educated student). This unfair acting may occur in any unproctored on-line exam. A specific type of these exams is where the answers cannot be ‘googled’ or shared via private chats or social networks, as the exam solution requires a good understanding of the topic. Such knowledge may involve a logical combination of available materials, maths calculation with custom inputs, and custom problem assignment.

Every student who accesses on-line course content, including the exam, leaves digital traces. These traces, in the form of on-line actions, are commonly archived by distance education systems. Our method uses these actions to indicate cheating. The indication is available after the exam has ended, or can be processed retrospectively several years to the past, based on the student’s data archival policy set by the educational institution.

Our method observes the on-line actions of the enrolled students in courses. For each student in a course, we define the exam session(s), i.e., the session where an exam was taken. The student’s speed of travel is calculated between two places, from which subsequent on-line actions were performed before and after the exam. The locations used are estimated using IP address geolocation. IP geolocation accuracy is limited and errors are possible [1,2]. In our method, we work with the location confidence area to avoid using uncertain locations. The speed of travel, size of location confidence area, area border to border distance, and other data are used to derive the student’s cheating risk score.

The method can be applied to any distance education system, where the data about students and their particular accesses to course on-line materials are logged and can be exported. Some of the current major distance education systems (also known as LMS (Learning Management Systems)) are Canvas LMS [3], Blackboard Learn [4], D2L Brightspace [5], Schoology [6], and Moodle [7]. We applied the method to the real students’ data from the Moodle distance education system, which is used by our university.

This work has a broad range of applications in on-line unproctored exams where knowledge about students’ cheating is needed. For example, depending on the course leader or educational institution policy, students indicated as cheating during an unproctored on-line exam may be immediately invited for a remote oral re-examination (over a video session) to validate their knowledge. It is always a possibility to invite a selected group of students (e.g., 5) to an on-line proctored re-examination, immediately after the unproctored on-line exam taken by all students (e.g., 200). This would certainly affect all students’ learning process, as it will deter other students from any dishonesty activity. The limited group of students for the proctored re-validation may be chosen randomly, or better, based on their cheating risk score, which we propose. It has to be noted that the knowledge re-validation has to be implemented in the course regulations, such as that a student will face a penalty, if the proctored exam result is significantly lower compared to the unproctored one. Unjustified nonappearance for re-examination also results in a penalty.

The students’ cheating indication can also be used in various social studies. One may compare the level of cheating in technical and non-technical courses, in different subjects (e.g., Maths vs. ICT), or across schools and universities. Research can provide further insight into age groups, gender, nationality, and grading systems.

In the paper, we also address the question of bypassing the method by students who want to cheat. A VPN or proxy connection may be used to mask their IP addresses in an attempt to avoid being caught when cheating. We elaborate possible scenarios with a conclusion that it is difficult to circumvent the method. We note that other anti-cheating measures in unproctored on-line exams have to be implemented as well for the protected system to work as a whole, such as the mandatory use of the Safe Exam Browser [8].

Another question we address are false cheating indications. A student may be associated with a cheating risk score, which is not correct. We discuss possible reasons behind this possibility. The first reason is errors in IP geolocation. In our method, we attempt to reduce those errors by a custom use of the IP address location confidence area. We further discuss the false indications caused by IP address changes due to general networking-related events, such as different assignments from the DHCP server. Our conclusion is that a set of rare conditions have to be met to produce a false cheating indication.

The paper is organized as follows. Related work is discussed in Section 2. In Section 3, we describe our method. This part deals with the input data, custom processing, cheating assumptions, and definition of the cheating risk score. In Section 4, we analyze the real students’ data from the Moodle distance education system. We present the results along with cheating examples. We also compare students’ cheating risk scores across academic terms. A discussion on bypassing the method and on false indications is covered in Section 5.

## 2. Related Work

The related work about unproctored on-line exams mainly deals with the exam content construction, analysis of students’ answer similarities, and notes about judicial validity. We have not found any related work dealing with the situation, when somebody else takes the exam instead of the legitimate student. To the best of our knowledge, this cheating issue is not explicitly discussed in the literature and no technical solution is provided.

COVID-19 has accelerated the adoption of on-line education. Most of the research addresses the teaching pedagogy and tailors the methods to the on-line environment. In [9], the authors have explored the challenges faced by educators to a sudden change to on-line learning due to the Covid pandemic. The qualitative research presented was based on interviews with 14 educators in a Singapore university. Some educators have replaced the invigilated exams by open-book exams with high-order questions. However, the educators showed concerns about assessment integrity and students’ dishonesty.

The authors of [10] recommend having continuous assessments rather than one large exam. They state that on-line exams (worth 40 % or more) can be used. However, identification of students is recommended for an on-line examination, either a part of the continuous assessment or the final exam. Identification is categorized into basic, medium, and high level corresponding to login/password, virtual face-to-face, and biometric, respectively. This would consume resources, and it is not scalable for large classes. Furthermore, the question bank should be large, question selection should be random, and answer time should be minimum to thwart an on-line search for answers.

Students’ unfair acting is also a problem in the Massive Open Online Courses (MOOCs). MOOC courses do not have a closed number of educational institution students and they are generally open for a very large number of students. The authors of [11] conducted an extensive survey on MOOC, particularly its development in the last decade. They analyzed 241 papers published between 2009 and 2019. The authors proposed six cognitive mapping dimensions and used them to discuss the state of the art of MOOC. They also elaborated five key questions; one of them was related to the students’ assessment. A particular point addressed was that there were concerns by the educators about verification of student identities who took on-line exams, and about plagiarism and other ways of cheating. Peer assessment and peer review were thoroughly discussed as an alternative to unproctored on-line assessments. In these assessments, students grade other students using instructions provided by the educators. These instructions may be modified by the students for better peer feedback.

In [12], the authors elaborated two techniques to analyse already taken on-line open-book exams to identify possible collusion. The techniques aimed to be processed retrospectively. First, they worked with the pairwise similarities of the answers. The Jaccard similarity index [13] was used to detect similarity in the answers. The authors stated that the Jaccard index’s heat maps could deliver a solid indication to inspect collusion by further manual validation. Second, they analyzed the student action log of the exam to reveal the parallel and leader-follower patterns. They focused on the time-series of questions and answers, and used a distance measure between them. The tasks that were not answered at least in one test were excluded. The results were presented for the selected pairs of students, where a high Jaccard index or similar answer timing suggested a suspicion of collusion.

The authors of [14] proposed an anti-collusion approach for on-line exams. They optimized the sequence of questions that are displayed to students in time synchronized slots. This extended the traditional techniques such as randomized order of questions, limiting the time, and having a large question pool. For example, a pool of 300 questions are needed for a 30-question test to have the average number of the same questions for two students below 3. The method reduced the pool of questions to a size of 1.5 times the number of questions in the test.

Authors in work [15] studied the results of 500 students in unproctored on-line exams taken in the Moodle distance learning system. The authors noted that there were no technological measures to significantly reduce cheating during on-line exams without breaking the student’s privacy. The final exam was taken in five rounds. The students in later rounds had better performance—the number of correct answers to the same questions from the earlier rounds was about 8% higher. The completion time for the same questions was ~18% shorter. The authors estimated that from 13 to 23% of the students cheated due to information flow between the rounds.

Any detected unfair acting during the on-line exam should be regarded as circumstantial evidence and not as a proof from the judicial point of view [12,16]. Furthermore, a suspicion of cheating during on-line exams can lead to suspension of degree granting until the issue is resolved [15]. On the other hand, students’ privacy should be considered when processing their data [17,18] for the cheating indication purpose.

## 3. Method for Cheating Indication

In the method proposed, we describe the input data, their processing, and definition of the cheating risk score. The input data consist of students’ actions. These are processed into learning and exam sessions. Student’s actions of particular interest are located by their IP addresses using a custom approach for the cheating indication. Next, the cheating indication event is defined and the student’s travel speed is calculated. Finally, the student’s cheating risk score is derived by elaboration of the assumptions related to cheating.

### 3.1. Students’ Actions and Sessions

During distance education, students regularly access the e-learning system with course curricula and other study materials published, including exams. Each access and data modification (e.g., course module display, file download, form filling, and exam item answered) is logged as a student’s action. The student’s actions form a stream of data. We note that we work with the student’s ‘actions’, not ‘logins’, in this context, as the system login itself by the ‘other’ student using the legitimate student’s credentials is not an act of academic dishonesty. Moreover, the login data do not provide information to indicate cheating in exams.

**Figure 1:**
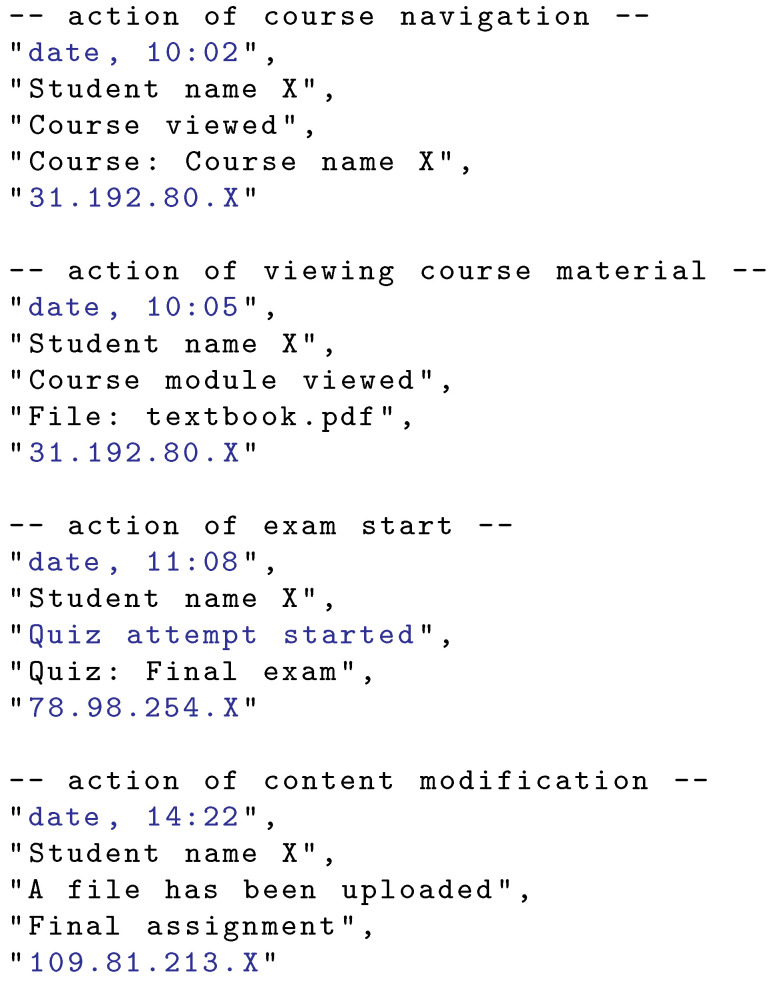
Student’s action general data. Items used in this work are highlighted.

Each student’s action consists of a set of data. General data, independent of the distance education system used, are shown in Figure 1. Such data commonly include date and time, student’s name, action name, action content, and student’s device (public) IP address. General actions covered in Figure 1 are (i) viewing the course landing web page, (ii) viewing a particular course material (pdf document), (iii) start of an exam in a form of a quiz (final exam), and (iv) file upload, which may be a photocopied hand-written text with the exam elaboration. The distance education systems provide the student’s actions in different formats and via different interfaces (e.g., web interface and REST API). Custom parsing may be needed to extract the relevant content, such as timestamps, IP addresses, and action names.

**Figure 2:**
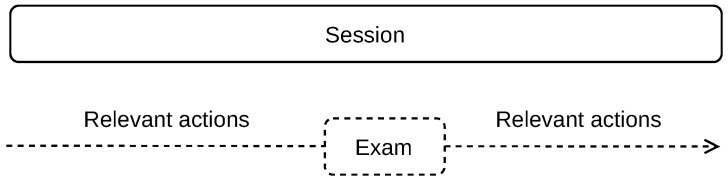
Relevant actions for student cheating indication are found before and after the exam within a session.

Our method is based on splitting the time-stamped student’s on-line actions into sessions. We are particularly interested in the actions before and after the exam, as shown in Figure 2. The exams are delimited by the specific action of exam start and the specific action of exam submission. Depending on the exam setup, particular answers to exam questions can be logged as one action or multiple actions, such as when each exam item is shown on a separate page. Our goal is to split the stream of student’s actions in the way that the relevant actions before and after the exam are contained in one session, without mixing separate exams into one session, or splitting one exam into multiple sessions.

**Figure 3:**
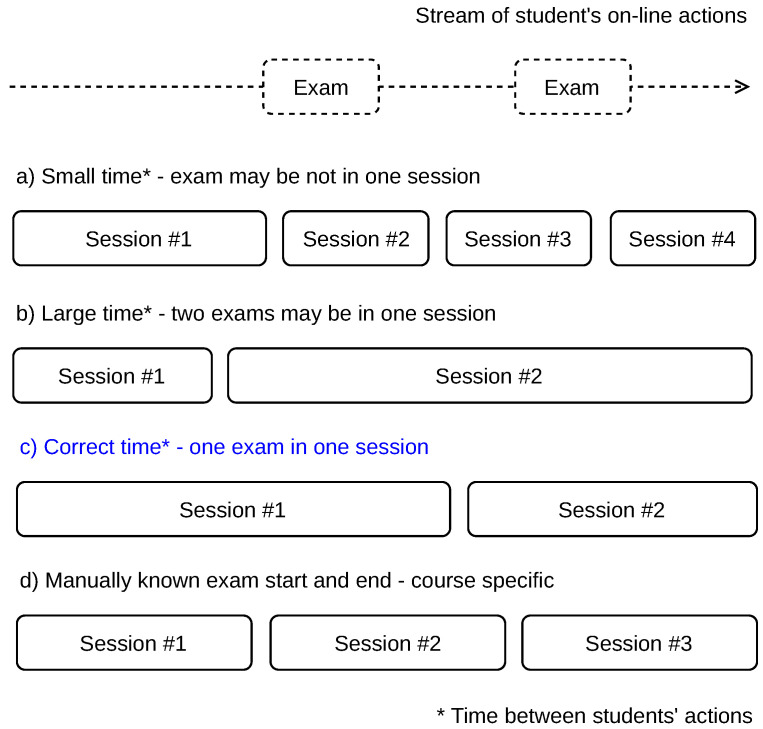
Splitting of student’s on-line action flow into sessions.

We elaborate four scenarios of the student’s action flow splitting into sessions. Figure 3 shows a stream of actions that includes two exams taken by a student (at the top of the figure). We use the time difference between two subsequent student’s actions to split the stream with the possible results described below.

- Scenario (a): A small time between actions is used and more sessions are delimited. A session may not contain all information related to cheating as the relevant actions before the exam, during the exam, and actions after the exam may be split into two or more sessions. This may happen when a student takes time to answer the exam items, especially when all exam items are displayed on a single page, or the time limit for the exam solution is long. Such a scenario may result in some cheatings not being indicated.
- Scenario (b): A large time between actions is used and less sessions are delimited. Two or more exams may be covered in one session, including all relevant information before and after each exam. The data related to separate exams are mixed this way, especially the actions between the exams. In this case, it cannot be differentiated which is the last action relevant to the first exam and which is the first action relevant to the second exam. Therefore, some cheating indications may be lost.
- Scenario (c): The optimal time between the actions is used to delimit the sessions. Such sessions include a proper set of relevant actions before the exam, actions during the exam, and a relevant set of actions after the exam. However, this optimal time is difficult to obtain as the number and time range of the relevant actions before and after the exam is not known and their number varies from case to case. In our method, we use this scenario to delimit the sessions.
- Scenario (d): The sessions are delimited by specific actions, which are manually found. For example, if the exam name is known, such as ‘Final exam—Course name X’ and the exam name is recorded along with the action name, which is ‘Attempt started’, such an action can be used to indicate the exam start. This, however, requires knowledge of the exam names, which is specific for each course. Moreover, it requires a manual inspection of all actions before and after the exam to find all relevant information. This scenario is therefore not suitable for automated processing.

Note that the student’s logins into a distance education system cannot be used for action flow splitting. The reason is that a student, either legitimate or the other, may login any time before the exam, login/logout several times within a short time, and logout anytime after the exam.

We use the time difference for action flow splitting (approximately) equal to the system inactivity logout time, which is derived from the distance education system used. This way, the delimited sessions contain the relevant actions before and after the exam, as we suppose that a student is not automatically logged out during an exam. A common inactivity logout time is one hour. An approximate time could be set in custom implementations to prevent the students from bypassing the method, as described in Section 5.

**Figure 4:**
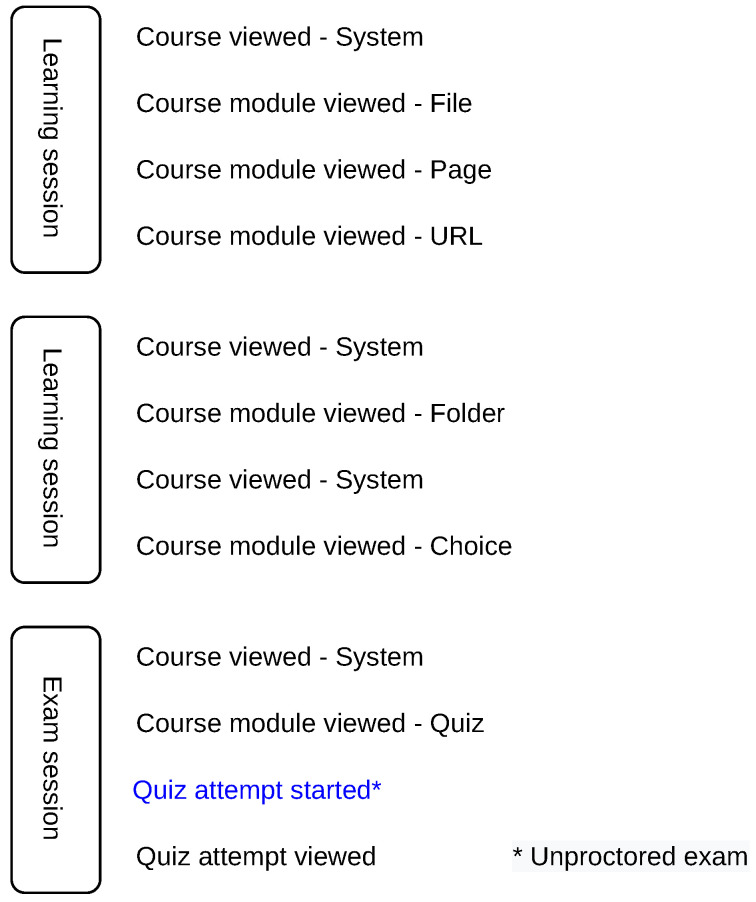
Learning and exam sessions. Exam sessions contain a general action for the exam start.

After the sessions are delimited, we identify the exam and learning sessions. Exam session is the one that contains a general exam start action (e.g., ‘Quiz attempt started’), which is independent of the course, exam name and exam setup (structure of the exam), as shown in Figure 4. For automated processing, the general action for exam start is derived from the distance education system used, which is shared across all courses in the education system. Other sessions are referred to as learning sessions.

### 3.2. Custom Location

The student’s on-line actions include the public IP address of the student’s device. We obtain the geographic location of the IP addresses. IP geolocation is commonly used by various Internet services and applications [19,20]. The location can be derived by looking up an IP geolocation database [21] or by active network measurements (typically latency) [22]. The active measurement delays the location process and cannot be used for large data processing, as we use in our method. We, therefore, customize the database-based approach for our cheating indication purpose.

IP geolocation has a limited accuracy [23,24] and there is active research ongoing [2,25] to improve it. Therefore, an IP address may not be accurately located and large errors are possible. For these reasons, the location confidence area may be returned by the geolocation database along with the location result in the form of latitude and longitude. Such an area, specified as a circle around the estimated location point, gives the information where the true IP address location is likely to be.

**Figure 5:**
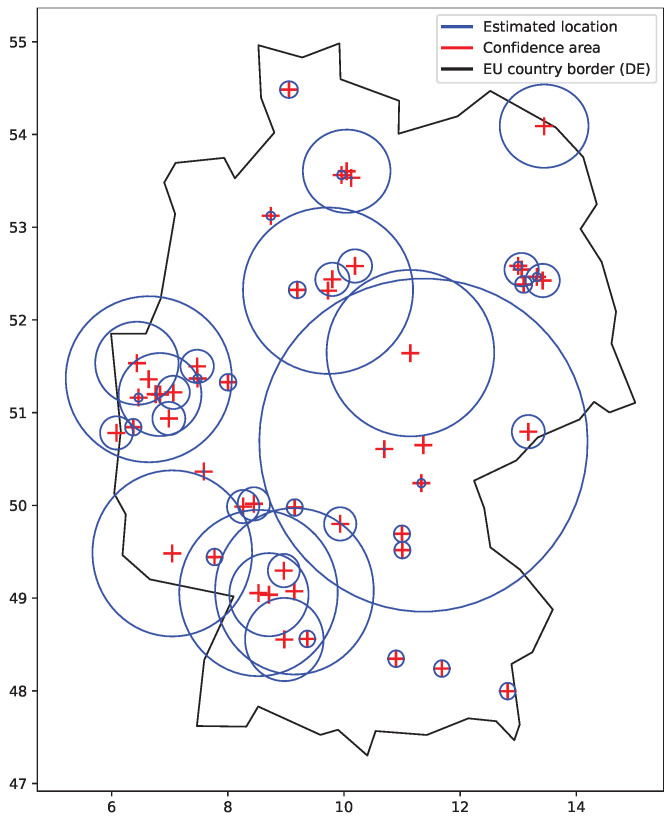
Confidence area and locations obtained for sample IP addresses. Large areas indicate uncertain locations. Small areas are used for trusted locations.

Figure 5 shows a sample of location confidence areas as circles that we observed for IP addresses of the RIPE Atlas [26]. The observed confidence areas were circles with radii of 1, 5, 10, 20, 50, 100, 200, 500, and 1000 km, which were obtained from the MaxMind GeoLite2 City database [27]. Different values may be observed for different location database providers. Typically, large confidence areas were returned for locations pointing to the country capital cities and country geographical centers, as these locations are used if there is not a better result available (stored in the geolocation database). However, many other returned locations also had a large confidence area. Smaller areas are used for the results where the returned location can be trusted, such those previously obtained via GPS or WiFi positions. The same locations and confidence areas may be shared by a range of addresses, defined by their network mask.

**Figure 6:**
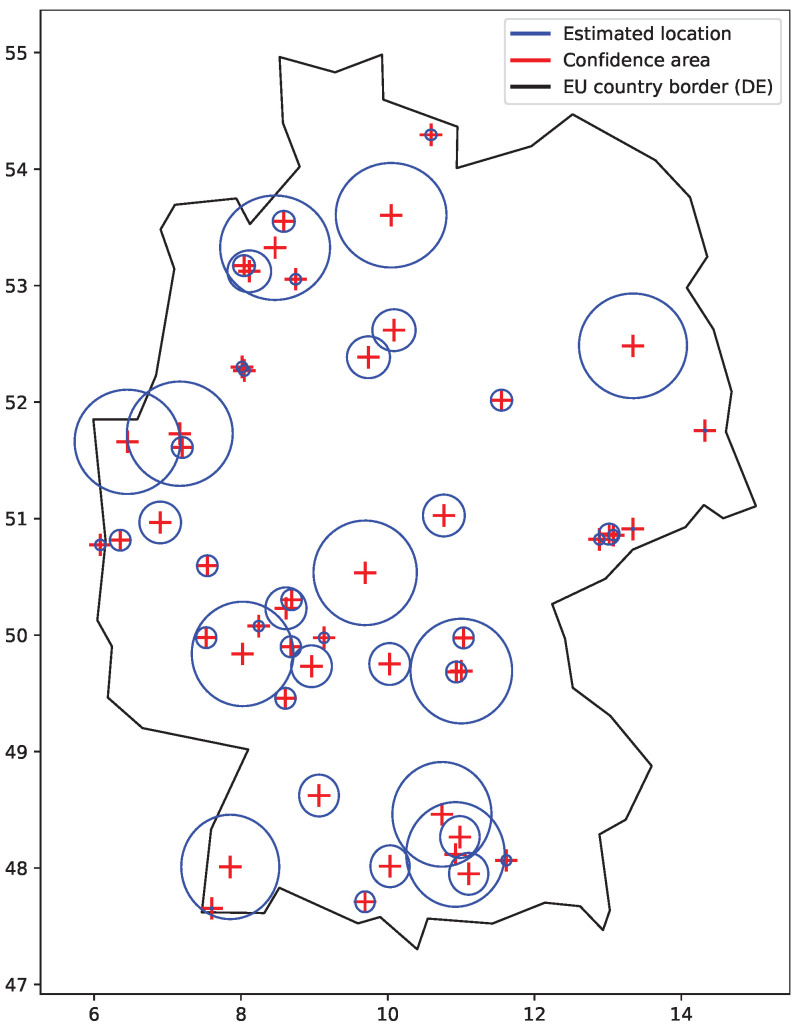
Confidence area and locations derived for sample IP addresses. Large areas are excluded and relatively small area boundaries are used within a country.

We remove (do not consider) uncertain locations with large confidence areas. This way we ‘lose’ some of the student’s actions, including the ones of particular interest. We, however, prefer a smaller number of indicated cheating cases to their larger number, which would include uncertain results. Figure 6 shows the situation when only smaller confidence areas are considered. The plot particularly shows that such confidence areas give relatively small boundaries to work with within a country or a neighboring group of countries.

**Figure 7:**
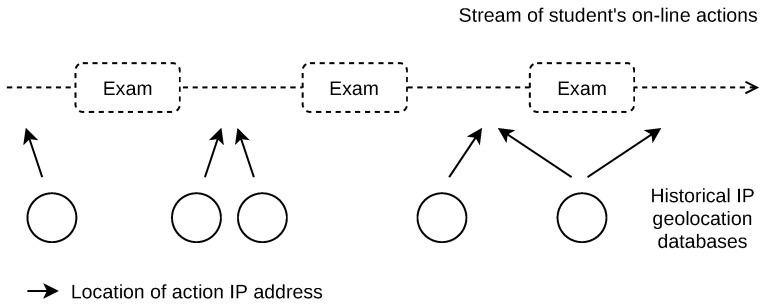
IP geolocation of student’s actions. Historical databases are used to eliminate errors due to date differences.

The use of database-based IP geolocation also allows us to derive the locations that were valid in the past, that is, the locations of IP addresses used in the past. This way, the cheating indication can be done retrospectively several years back, depending on the availability of the archives of the student’s actions. However, the location error increases when the geolocation database release date and the date of the students’ action do not match (e.g., the action date is older than the database build date). We, therefore, locate the students’ actions using a set of historical geolocation databases. The database with the closest date match to the student’s action date is used, as shown in Figure 7.

### 3.3. Travel Speed

**Figure 8:**
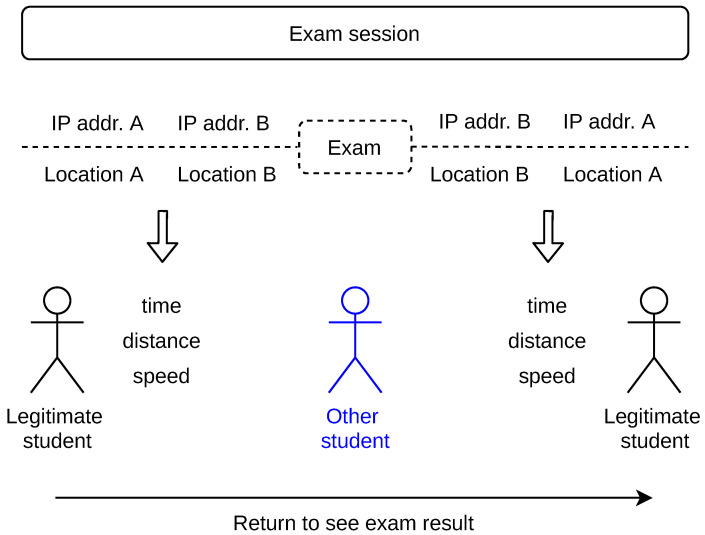
Indication events during exam session. The case of two student’s role changes is shown.

We calculate the student’s speed of travel during the exam sessions, as shown in Figure 8. The speed is calculated on the occasion when the IP address changes between two subsequent actions. We further refer to these occasions as ‘indication events’. An indication event presumes that the students possibly changed roles, that is, the other student now acts as the legitimate or vice versa. Such possibility is further elaborated in Section 3.4. The indication events may occur (i) before exam, (ii) after exam, or (iii) both before and after exam. The latter may happen when the legitimate student is curious to know the result of the exam taken by the other student, immediately after the exam ends. In Figure 8, we refer to this scenario as ‘Return to see exam result’. Multiple indication events during an exam session are also possible when the legitimate and the other student work in the education system simultaneously.

### 3.4. Cheating Risk Score

We assess each student taking an exam by a cheating risk score. The risk score is based on an elaboration of a set of assumptions related to cheating: distance, travel speed, location area, and the number of indication events.

**Figure 9:**
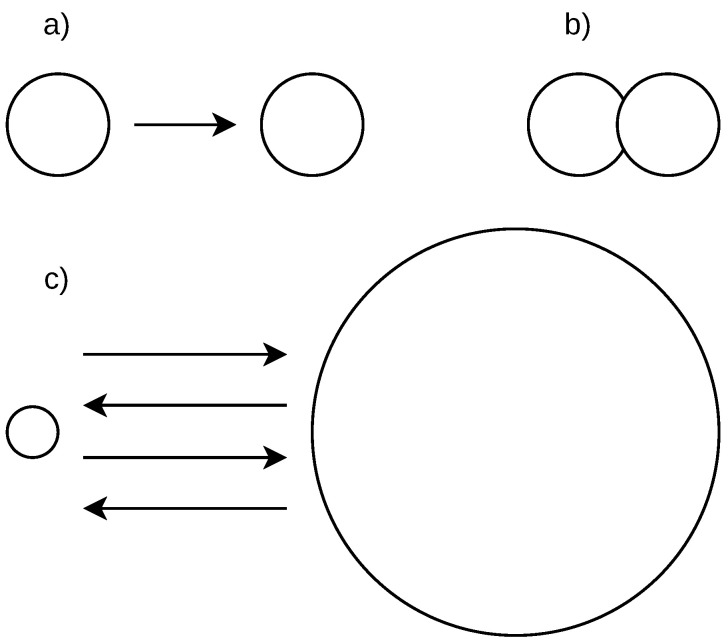
Confidence areas related to indication events: (**a**) border to border distance, (**b**) risk score is zero, and (**c**) multiple indication events and use of a larger area.

- Distance—Distance is measured for each indication event as the closest border–border distance of the two location confidence areas, as shown in Figure 9 (case a). If the circles intersect, we set the distance and, consequently, the risk score as zero (case b). Our reason is that the two locations estimated for different action IP addresses may be actually the same. A higher area border–border distance increases the score.
- Travel speed—Student’s speed of travel is calculated for each indication event given the time between the subsequent actions and the distance between the locations of action IP addresses. A higher speed increases the score.
- Area size—Area size is expressed as the circle radius. For each indication event, we consider the larger radius of the two confidence areas. Large radius decreases the score. Our reason is that larger areas are used for less trusted locations. We note that larger areas are not considered at all as they are removed from the processing at the previous location step, as described in Section 3.2.
- Indication events—An exam session may have a number of indication events. Each indication event produces a risk score. If the students change roles only once during the exam session, the risk score is calculated from one indication event. In the case of a legitimate user returning to the education system to see the exam result, which the other student took, there are two indication events and the resulting risk scores are summed (the student is ‘caught’ twice), as shown in Figure 8. In the case of the legitimate and the other students changing roles multiple times within the exam session (the student is ‘caught’ multiple times), as shown in Figure 9 case (c), all cheating risk scores are summed. A student with more indication events during an exam session with lower risk scores can obtain a greater summed risk score than a student with one indication event with a high score.

By implementing the above assumptions into an equation, we define the cheating risk score *r* for a student taking the exam in an exam session as

(1) $$r=\sum_{i=0}^{N}\frac{s_{i}\times b_{i}}{\max\{C_{x,i},C_{x,i}\}}\;,\;\;\;b_{i}=0\;\mathrm{if}\;C_{x,i}\cap C_{y,i}\neq ⌀$$

where i=1,⋯,N is the indication event, *N* is the number of indication events in an exam session, $s_{i}$ is the student travel speed for an indication event *i*, $C_{x,i}$ is the confidence area radius of the first location of the indication event, $C_{y,i}$ is the confidence area radius of the second location of the indication event, and $b_{i}$ is the border to border distance of $C_{x,i}$ and $C_{y,i}$, which is zero if the areas intersect.

The risk score may not be normalized. The length of the exam sessions is not limited and thus the maximum number of indication events per session is unknown. The risk score may be calculated for a single exam session or summed up for multiple exam sessions (e.g., mid and final exams) in a course, or the whole study program with many courses and exams.

## 4. Application to Students’ Data

We applied the method to the real students’ data and present the cheating occurrences with examples.

### 4.1. Courses and Students

**Figure 10:**
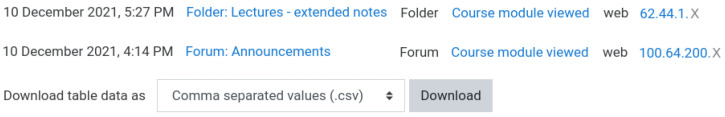
Export of students’ actions from Moodle. The figure is modified for presentation and privacy reasons.

The student’s data come from the courses that were taught during the COVID-19 pandemic, when the university was closed for attendance education. It was up to the course leaders to choose the form of on-line examinations, such as unproctored, proctored with a mandatory web camera showing the student face, or oral examination over a video session. We worked with the data of the students enrolled in selected courses, where the on-line exams were unproctored. For comparison reasons, we also included courses with on-line unproctored exams that were taught before and after the university was closed for attendance education, i.e., unproctored on-line examination was a regular part of the courses. We exported all students’ actions for each course. The export was done from the Moodle distance education system via the web interface [28], which is in a modified form shown in Figure 10. Actions with unusable data, such as carrier-grade NAT IP addresses, were excluded.

Table 1 overviews the students’ data. In total, 22 courses were considered. Majority of the courses (20) were taught in the academic year 2020/2021, either in winter or summer terms. The unproctored on-line examination as a result of restrictions due to the Covid pandemic was processed in the winter term of 2020/2021 and summer term of 2019/2020. Each semester duration was 13 weeks. The start date of the first course was 9/2019. The end date of the last courses was 6/2021.

The table shows the number of sessions detected using a one-hour difference between the subsequent actions. A noticeable point is that the students used a low number of IP addresses during the courses (4), presumably as a result of the reduced mobility during the country lockdown.

**Figure 11:**
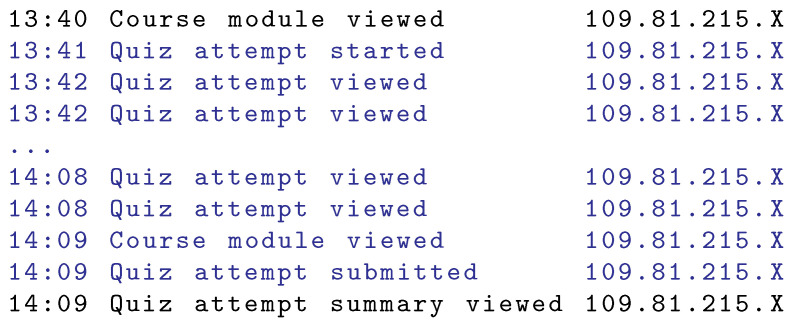
Sample of an exam session. Exam type with each question on a separate page is shown.

A sample exam session is shown in Figure 11. Depicted is the exam type where each question is listed on a separate page. The exam took 28 minutes as shown by the actions named as ‘Quiz attempt started’ and ‘Quiz attempt submitted’. The transition from page to page (move to the next question) generated the action named as ‘Quiz attempt viewed’.

We found 659 IP addresses associated with indication events in exam sessions. We located these IP addresses using 58 MaxMind GeoLite2 City geolocation databases. The first database build date was 9/2019, the last database was dated to 6/2021. The mean date difference between the databases was 11 days. For each IP address location, the closest database in terms of its build date to the student’s action date was used, as shown in Figure 7. The mean difference between the database dates used for the location and student’s action dates was 4 days.

**Figure 12:**
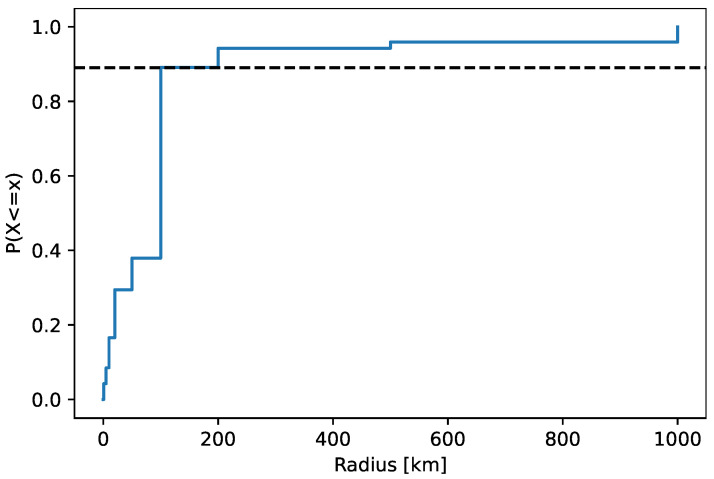
Cumulative distribution function of IP address confidence location area radius in indication events.

Figure 12 plots the confidence radius of the located IP addresses related to indication events. The median radius was 100 km. The cumulative distribution function F(x)=P(X≤x) of the radius has a tail that consists of ~10% of the locations, as highlighted in the figure. We removed the tail locations from further processing of cheating indication, as it is described in Section 3.2.

### 4.2. Cheating Examples

We show in detail an example of cheating that covers multiple indication events. The cheating risk score is also calculated for the example exam session. We also show a geographical plot of other indicated cheatings.

**Figure 13:**
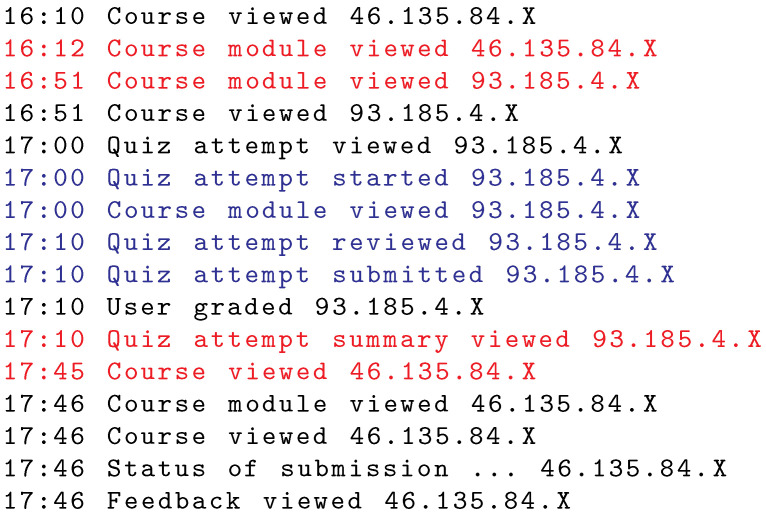
Example of an exam session (selected part) with indicated cheating. Indication events occurred before and after the exam.

Cheating that covers multiple indication events may occur when the legitimate student is interested to see the exam result, previously taken by the other student. A list of subsequent actions of this case is shown in Figure 13. The first action of the indication event before the exam occurred at 16:12 and the second action at 16:51. This is when the students first changed the roles. The exam listed had all questions on a single page as there were no actions related to page transition. In particular, the exam is a partial midterm exam with a short time limit for its completion. The second indication event occurred at 17:10 (first action) and 17:45 (second action), i.e., when the students changed roles for the second time after the exam.

Table 2 shows additional data of the two indication events. The content of the actions is shown, including the exam name and course name. The same city names and locations indicate the return of the legitimate user. The distance between action places was 139 km and the student’s travel speed was 214 and 240 km/h, respectively. The location confidence areas were different. However, calculation of cheating risk score considers only the larger area, which was 100 km. The border to border distance of the two confidence areas was 38 km. The cheating risk score is (N=2)

(2) $$r=\sum_{i=0}^{N}\frac{s_{i}\times b_{i}}{\max\{C_{x,i},C_{x,i}\}}=\frac{214\times 38}{100}+\frac{240\times 38}{100}=173\;.$$

We note that, in general, there may be more indication events within an exam session, especially in cases when the legitimate and the other student alternate accesses.

**Figure 14:**
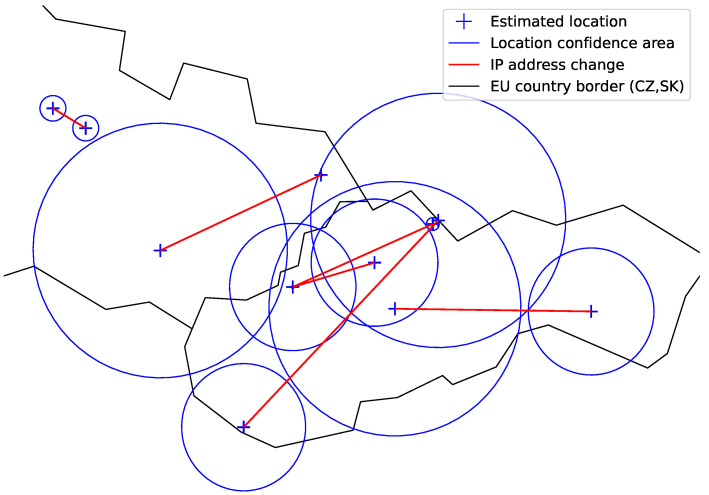
Geographical plot of selected indicated cheatings. Plotted are location confidence areas and distances associated with indication events.

There were in total 145 exam sessions with indication events detected. A geographical plot of sample indication events is shown in Figure 14. The red line between the confidence location areas links the actions of the same indication event. Plotted are only the events with a positive border to border distance. Indication events with the confidence area of the IP address location greater than 100 km (radius) were excluded to avoid working with uncertain locations.

### 4.3. Students Selection for Re-Examination

**Figure 15:**
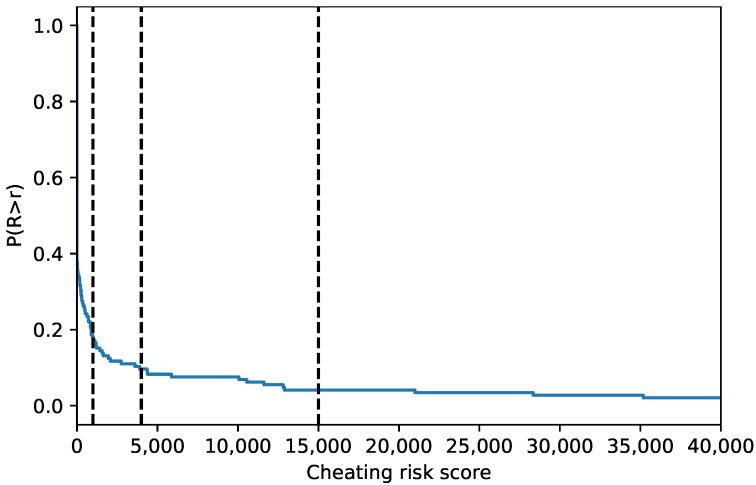
Survival function of cheating risk score.

Figure 15 shows the cheating risk score for all students in 22 courses evaluated. The score is calculated per exam session using Equation (1). The survival curve plotted

(3) $$S\left(r\right)=P\left(R>r\right)=1-F\left(r\right)=\int_{r}^{\infty }f\left(u\right)du$$

gives the probability of a student’s cheating risk score greater than *r*, *R* is the random variable expressing the risk score, and F(r)=P(R≤r) is the cumulative distribution function. The survival function has a sharp drop with a flat tail. The tail shows that only a few students had a large cheating risk score of ~15,000 and greater.

Based on the data collected, the cheating risk score can be used to select the student for re-examination. For example, if we want to select 20% of the students, we may re-examine the students with a cheating risk score greater than 1000 (approximately). Similarly, to select 10% of the students, the cheating risk score greater than 4000 can be used. To select only students with a firmer indication of cheating, the score greater than 15,000 can be used.

### 4.4. Cheating Comparison

The cheating risk score can be used to compare the students in terms of cheating, as listed in Section 1. We compare the students by restricted mean survival (RMS)

(4) $$\mathrm{RMS}\left(r\right)=\int_{0}^{r}S\left(u\right)du\;,$$

which calculates the area under the survival curve up to the risk *r*. The restriction *r* (upper limit of the integral) is used to avoid the inclusion of the long curve tail as this may have a strong effect on the integral. The value of *r* may be selected based on the risk scores calculated.

**Figure 16:**
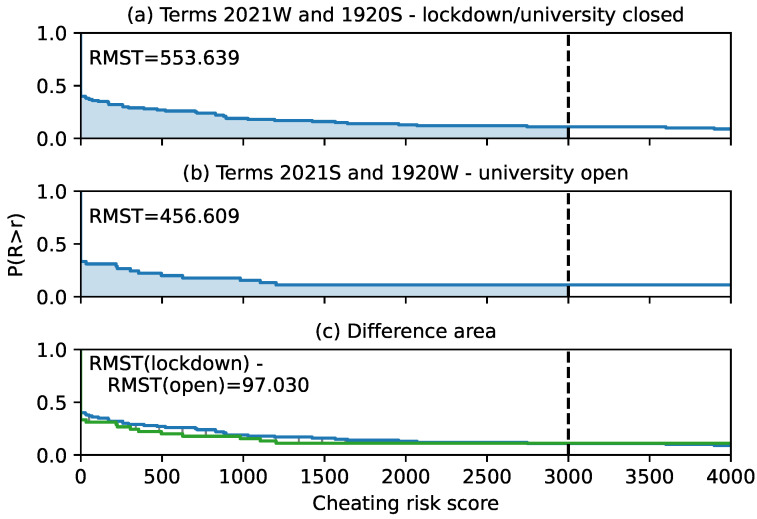
Comparison of student cheating risk score between four academic terms, two affected and two unaffected by the lockdown due to COVID-19 pandemic. W—winter term; S—summer term.

Figure 16 compares student cheating risk scores in courses taught in four academic terms. Two of the terms, the winter term of 2020/2021 and the summer term of 2019/2020, were affected by the country lockdown due to COVID-19 pandemic. The university was closed for attendance education and all exams were distant, including unproctored on-line exams in the form of a quiz. Two other terms, summer 2020/2021 and winter 2019/2020, were unaffected and the students attended the classes. The courses in these terms had unproctored on-line exams on a regular basis, that is, independent of the lockdown. The difference in the delimited area between the survival curves was calculated by [29] and was ~97, indicating that the students in question cheated more in the terms affected by the COVID-19 pandemic, but the difference was not significant.

## 5. Discussion on Bypassing the Method and False Cheating Indication

We discuss whether students can avoid being indicated as cheating. We also elaborate the possibility of false cheating indications.

### 5.1. Bypassing the Method

Students, who are aware of the method, may use a VPN connection (or a web proxy connection) to mask their addresses when cheating. We, however, show that it is difficult to bypass our method, thus not worth taking the risk to cheat. We present two scenarios of VPN usage: naive and better.

- Naive—In this scenario, the other student wants to bypass the cheating indication. He/she uses a VPN connection to mask the IP address of the relevant actions before and/or after the exam. The IP address of the VPN server will be, however, processed the same way as the genuine IP address of the other student. That is, an indication event will be detected and a cheating risk score for the legitimate student will be calculated. In this case, the detection will depend on the geographical location of the VPN server and its distance to the legitimate student. It is likely that the risk score will be even higher, as the VPN server may be located in a different country or even continent, than the legitimate student. There is an option to use a VPN server that is geographically close to the legitimate student, provided there is any available and, also, provided the student knows how the confidence area is handled.
- Better—In this scenario, both students agree and synchronize themselves in an attempt to bypass the cheating indication. They both use the same VPN server to mask their IP addresses for all relevant actions before and/or after the exam. This, however, requires the knowledge of the method implementation details as they need to ‘properly’ guess when the relevant actions start before the exam and when the relevant actions end after the exam. The time used for the student’s action flow splitting may be different by implementation and thus not guessable by the students. A student’s guess may reduce the risk of being indicated as cheating, but it is not bypassing the method.

We note that other anti-cheating measures in unproctored on-line exams have to be implemented as well for the protected system to work as a whole. For example, it is the mandatory use of the Safe Exam Browser [8], which blocks the desktop sharing. Furthermore, legitimate students’ presence in the system during the exam session may be enforced by a course regulation of possible random web-camera face check, requested by communication in the distance education system.

Student’s use of a VPN to mask his/her IP address during an exam session for other reasons not related to cheating, e.g., VPN activation by mistake, will result in false cheating indication. Please consult our statement at the end of Section 5.2.

### 5.2. False Cheating Indication

Some networking-related events may cause the IP address of the student’s device to change. This change may be misinterpreted in the cheating risk score calculation. Furthermore, false cheating indications may be caused by errors in IP geolocation.

In general, IP addresses change due to their different assignments from the DHCP server, if dynamically allocated. The assignment of a different IP address may occur when the device boots up. It may also occur when the device is booted, such as if the IP address is renewed manually or its lease time expires. Another reason for an IP address change, which may occur at mobile devices, is when the Internet connection is switched between two networking interfaces, e.g., WiFi and the data plane. IP addresses of mobile devices also change when they are moved from a network to another and they obtain IP configuration from a different DHCP server. IP address may also be changed manually.

We consider these assumptions related to our method:

- The common (not cheating related) IP address changes occur independently of the time of our interest, that is, during exam sessions. Considering the likelihood that an IP address change occurs in this time in relation to the rate of IP address changes caused by ISPs [30], we assume it is a rare situation.
- We further assume that students typically: (i) do not use mobile devices for taking the exams, (ii) do not manually renew or change IP addresses during exam sessions, and (iii) do not move from one network to another with a different DHCP server during exam sessions.

If such a rare situation occurs and a new IP address is assigned to the student’s device during the exam session, an indication event is detected by the method. In this case, the cheating risk score is calculated with two possible outcomes:

- Zero value (cheating indication not applicable)—The cheating risk score of zero value will be calculated if the new assigned IP address and the previous IP address are geolocated to the same or close location. In this case, the location confidence areas will intersect, as shown in Figure 9 case (b), and the result of Equation (1) is zero.
- Positive value—A false cheating risk score will be calculated when the new IP address is not geolocated to the same location as the previous one and there is a large distance between them.

Last but not least, we again note that the IP geolocation has errors [1], including the location results with smaller confidence areas, thus false cheating risk scores may be calculated for this reason.

Based on this discussion, we state that our cheating risk score is informative and may not be used as evidence, as it is also discussed in Section 2. Our suggested use is in students’ selection for knowledge re-validation and for comparison purposes. Higher credibility may be given to the cases when high cheating risk scores repeat for certain students in different courses or over years. It may be also combined with other sources of cheating indications to deliver firmer conclusions.

## 6. Conclusions

Distance learning has provided education to students in the uncertain Covid era. However, less thought is given to the cheating issue in the on-line unproctored exams. We addressed the cheating scenario when somebody else takes the exam and acts as a legitimate student.

Our method is based on analysis of the traces the students leave when they access the course on-line content. The distance education systems commonly log these traces. The method indicates the cheating right after the exam and, also, several years to the past (based on the log archive availability). We applied the method to the selected courses where the examination was unproctored. We also compared the difference between the academic terms affected (country lockdown) and unaffected by the COVID-19 pandemic.

Several applications of this work were elaborated. The students with the highest cheating score can be invited for an oral re-examination (over a video session) for knowledge verification. Such a previously announced re-examination, even for a limited number of students, would positively affect all students when they prepare for the exam. Besides these straightforward applications, the cheating risk score may be used for statistical and comparison purposes, such as behavioral and social studies.

The work is solely based on data accessible by the teachers or distance education system administrators.

## Figures and Tables

**Table 1:** Data about 22 courses processed for students’ cheating indication.

Students	Actions	All Sessions	Exam Sessions	IP addr.	IP addr/st/crs *
3649	1,609,770	234,399	7674	20,042	4

* Median of used IP addresses per student per course.

**Table 2:** Student’s actions related to cheating example. The case of returning legitimate user is shown.

Time	IP Address	Action Content	Lat, Lon	City
16:12	46.135.84.X	Quiz: Test LC (MO_17)	49.1500, 16.6167	Brno
16:51	93.185.4.X	Course: Course X	49.6833, 18.3500	Frydek-Mistek
17:10	93.185.4.X	Quiz: Test LC (MO_17)	49.6833, 18.3500	Frydek-Mistek
17:45	46.135.84.X	Course: Course X	49.1500, 16.6167	Brno
